# Vertical distributions of dolphinfish (*Coryphaena hippurus*) in the Eastern Pacific Ocean suggest variability in potential associations with floating objects

**DOI:** 10.1371/journal.pone.0276873

**Published:** 2022-11-01

**Authors:** Sofia Ortega-Garcia, Christopher R. Perle, Nicholas M. Whitney, Ruben Rodriguez-Sanchez, John O’Sullivan, Stephanie Snyder Koch

**Affiliations:** 1 Instituto Politécnico Nacional-Centro Interdisciplinario de Ciencias Marinas, La Paz, B.C.S., Mexico; 2 Florida State College at Jacksonville, Jacksonville, FL, United States of America; 3 Anderson Cabot Center for Ocean Life, New England Aquarium, Boston, MA, United States of America; 4 Newport Aquarium, Newport, KY, United States of America; 5 Monterey Bay Aquarium, Monterey, CA, United States of America; 6 Thomas More University, Crestview Hills, KY, United States of America; Hawaii Pacific University, UNITED STATES

## Abstract

Floating objects play a pivotal role in pelagic ecosystems by serving as shelters, meeting points, cleaning stations, nurseries, and feeding grounds. The abundance of these objects is increasing globally in the form of flotsam, plastics, discarded or lost fishing gear, and fish aggregating devices (FADs) deployed by commercial fisheries. However, it is difficult to measure how often and in what ways fish interact with floating objects in pelagic environments. Dolphinfish (*Coryphaena hippurus*) is prevalent among the fish species that associate with floating objects, but the extent to which dolphinfish utilize them is unclear. This study applies existing knowledge of FAD-associated dolphinfish diving behavior to identify periods of potential association with floating objects in a remote telemetry dataset of 23 fish with a total of 678 days at liberty spanning two distinct regions within the Eastern Pacific Ocean. Fish inhabiting waters off the western coast of Baja California Sur, Mexico spent significantly more time exhibiting behavior indicative of association with floating objects than those off the coast of Oaxaca, Mexico. When not exhibiting this behavior, dolphinfish in both regions occupied similar vertical habitats, with western Baja fish utilizing more of the water column than Oaxaca fish. Observed regional differences in behavior were coincident with regional differences in size (Oaxaca fish fork lengths ranged from 103 to 118 cm (mean = 110 cm), while Baja fish ranged from 85 to 106 cm (mean = 93 cm)). Although larger fish in the Baja region displayed behavior consistent with smaller Baja fish, future studies should investigate whether the observed regional differences are due to (i) size, (ii) sex, (iii) oceanography, or (iv) availability of floating objects. Dolphinfish are an important mid-trophic level predator and potentially sustainable fishery resource. Understanding their behavior and use of floating objects is of both ecological and economic importance–particularly in the context of expanding international FAD-based fisheries. Our study suggests dolphinfish spend a large amount of their time exhibiting potential floating object associated behavior, and this could influence their population structure and growth.

## Introduction

In the open ocean, floating objects can develop into entire ecosystems by attracting species ranging from plankton to top predators (including humans) [1–5]. These floating objects range from natural debris such as sargassum, kelp, and logs, to man-made fish aggregating devices (FADs, description in [1]) and discarded plastic [6,7]. While research has revealed an overall ecological significance of floating objects [2,3,5] and high catch rates around FADs have increased their use by fishers [8–11], the extent to which individual fish utilize floating objects throughout their lifetimes remains unclear [12]. This study uses remote telemetry data to describe the vertical movements, frequency, and timing of behaviors consistent with previously reported FAD associations of dolphinfish (*Coryphaena hippurus*), one of the most abundant fishes observed at floating objects around the world [3,4,9,12].

With its circumtropical distribution (limited to sea surface temperatures between 19°C–31°C; [13]), early maturation (within the first year; [14,15]), fast growth rates [15] and high food value [16], dolphinfish have the potential to serve as a sustainable source of protein for a growing human population [17]. Dolphinfish comprise a large component of bycatch in the worldwide purse seine fisheries [1,4,5,8,18,19]. In the Eastern Pacific Ocean (EPO), dolphinfish are targeted in commercial longline, sport and artisanal fisheries [20–23] and landed incidentally in the purse-seine fishery mostly during sets associated with floating objects–either DFADs (drifting fish aggregating devices) or log sets (sets on any natural floating object, often algae or logs) [24].

Tracking studies have provided insights into the behavior of dolphinfish at known FADs [25,26], but little is known about the frequency, distribution, and ontogenetic patterns of its association with floating objects in general. Diving behavior of dolphinfish has been shown to be highly surface oriented [13,27,28]; however, dolphinfish are known to shift diving behavior according to whether or not they are associated with a FAD [26]. Over tracking intervals of hours to several days, Whitney et al. [26] found dolphinfish occupied unique depth distributions when associated with observed FADs versus when not. In the Pacific and Indian Oceans, dolphinfish have been shown to dive significantly deeper and have a more variable vertical distribution when not associated with a FAD than when associated with one [26]. Furthermore, when individuals were tracked for short periods of time, FAD associated dolphinfish were more likely to have deeper excursions during the day than at night, while the reverse was true when fish were not associated with FADs [26]. Study region and monitoring methods had no significant effect on behavioral pattern, suggesting that remotely observed vertical behavior could be used to delineate whether or not dolphinfish were associated with floating objects throughout their range, without explicit knowledge of the actual presence of the objects [26].

Following this logic, we apply the distinguishable vertical distributions of dolphinfish observed by Whitney et al. [26] both at FADs and away from them to a remote telemetry dataset collected in two distinct regions of the Eastern Pacific Ocean (EPO) to identify periods of time when dolphinfish were potentially associated with floating objects. By comparing the frequency, timing, and variability of modeled floating-object-associated behavior between regions, we can better understand the ontogenetic, regional, and oceanographic forcings on ecosystem dynamics and fishery interactions of dolphinfish.

## Materials and methods

## Study area

**Fig 1:**
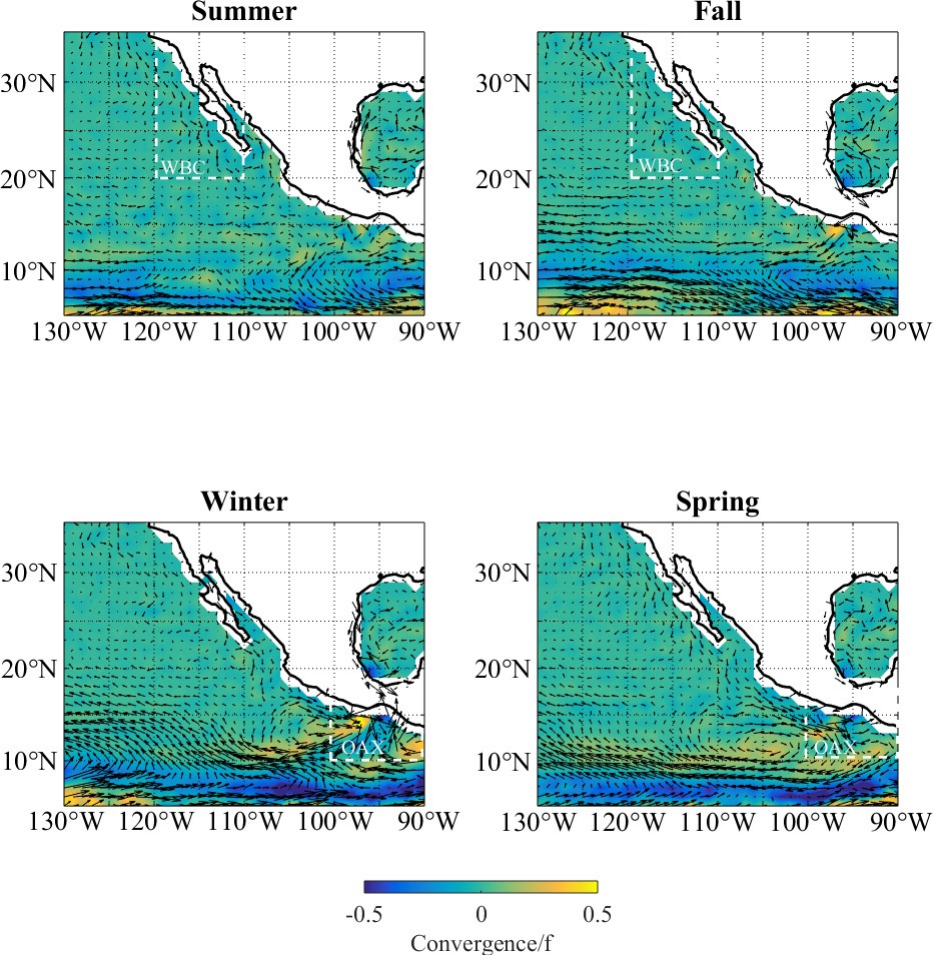
Seasonal patterns of currents (represented by black arrows) and convergence (represented by color) in the study region. Boxes denote the spatial extent of the tagging data in each region. The California Current strengthens along Baja in summer bringing kelp into the WBC region; while currents in summer through fall and the rainy season bring logs from the river deltas in Central America to the OAX region in the winter months. Tagged fish were available in WBC in summer and fall; while fish were available in winter and spring in OAX.

Tags were deployed on dolphinfish in two distant and distinct Mexican regions of the Eastern Pacific Ocean (EPO, as delineated by Harley 2015 [29]). A full description of tag deployment and geolocation processing methodology can be found in Perle et al. [13]. Briefly, “Northern” tagging locations within Mexican waters were west of the Baja California Peninsula (WBC), while “southern” tagging locations occurred in the coastal waters west of Oaxaca, Mexico (OAX, Table 1). Tagging locations were selected due to their accessibility to the authors given international, logistical and financial constraints. Tagged dolphinfish throughout their time at liberty were roughly bounded by 20–35°N, 110–118°W in the WBC and 10–18°N, 85–100°W in OAX (Fig 1). Floating macroalgal mats and logs are supplied by the kelp forests of the California Current System and the tropical rainforests of Central and South America and distributed throughout both the regions by local currents (Fig 1) [30,31]. These floating objects along with drifting FADs (man-made fish aggregating devices) are targeted by local fisheries, including, but not limited to, the tropical tuna purse-sein fishery [3].

**Table 1:** Description of tagging data utilized in this study with total deployment duration reflected in the dates at liberty. Sample sizes for diurnal and 24-hr periods reflect the number of periods with sufficient data to classify behavior.

Region	Fork Length (cm)	Sex	Dates at Liberty	Diurnal Periods (Days, Nights)	24 hr Periods
WBC	85	Female	9/24–11/26, 2018	59 (27, 32)	11
WBC	88	Male	7/10–7/22, 2017	25 (12, 13)	12
WBC	89	Female	9/21–10/4, 2018	25 (13, 12)	11
WBC	90	Female	10/3–10/26, 2010	46 (23, 23)	22
WBC	90	Male	7/21–7/27, 2013	14 (7, 7)	7
WBC	91	Male	10/4–10/15, 2010	24 (12, 12)	12
WBC	93	Female	10/3–10/30, 2010	51 (24, 27)	23
WBC	93	Male	7/21–7/27, 2013	14 (7, 7)	7
WBC	94	Female	9/28–12/5, 2017	49 (24, 25)	11
WBC	95	Female	10/4–10/11, 2010	16 (8, 8)	8
WBC	95	Female	9/24–10/26, 2019	60 (28, 32)	29
WBC	98	Female	7/23–9/1, 2013	45 (22, 23)	21
WBC	100	Male	7/21–9/14, 2013	58 (30, 28)	16
WBC	106	Male	7/23–8/14, 2013	17 (9, 8)	7
OAX	103	Male	2/12–4/1, 2014	76 (39, 37)	26
OAX	103	Male	1/16–1/27, 2020	23 (12, 11)	12
OAX	107	Male	2/12–3/25, 2014	67 (37, 30)	29
OAX	108	Female	1/17–2/22, 2020	55 (29, 26)	23
OAX	110	Male	1/17–2/29, 2020	79 (37, 42)	30
OAX	110	Male	1/17–1/24, 2020	15 (8, 7)	8
OAX	113	Female	2/12–4/5, 2014	72 (41, 31)	27
OAX	115	Female	1/17–1/28, 2020	23 (12, 11)	12
OAX	118	Male	2/12–3/21, 2014	62 (31, 31)	25

Although close in proximity and connected by regional currents, the two regions present dolphinfish significantly different physical environments. The sea surface temperatures of the WBC region vary seasonally (average temperatures ranging from approximately 18°C in the months of Feb-Apr to 24°C in Aug-Sep) and are cooler than those of the OAX region (average sea surface temperatures remain approximately 26–28°C throughout the year) [13]. Vertical characteristics of the water column also differ. The isothermal depth (i.e., the maximum depth at which the temperature remains within 0.8°C of the average temperature of the upper 5 m) is significantly deeper in the WBC than in the OAX [13]. The oxygen minimum zone (OMZ) is also deeper in the WBC than in the OAX [32].

### Tagging methodology

Dolphinfish were tagged during the Monterey Bay Aquarium’s Animal Care Division field collection trips via long haul sportfishing vessel R/V Shogun in WBC and small artisanal fishing pangas in OAX. Fishing method varied between the two regions–rod and reel (WBC) and longline (OAX); however, tag models and tagging techniques were largely consistent across regions (Complete details of tagging protocols are described in Perle et al. [13]). All fish were tagged under authority of the Comisión Nacional de Pesca y Acuacultura permit numbers DAPA/2/130910/ 044423, DAPA/2/030511/01246, and DGOPA- DAPA-01595/13. All fish were tagged with electronic pop-up satellite tags (PSAT; MiniPAT, Wildlife Computers Inc., Redmond, WA, USA). Fork length and sex were observed and reported prior to release (Table 1). In both regions, only dolphinfish with fork lengths greater than 80 cm were targeted and selected for tagging.

All tags were programmed to release from fish after either 60 or 90 days; resulting deployments shorter than one week were excluded from this study. During deployment, the PSATs recorded depth, water temperature, and light at 3 s intervals. These data were then algorithmically subsampled, and summarized onboard the tags, then transmitted to satellites and stored in the Wildlife Computers® data portal [33].

Vertical behavior modeling reported here relied only on the received subsamples of timeseries data. The subsampled, transmitted and received data of depth and water temperature provided incomplete timeseries at sampling intervals of 2.5 min. Twelve-hour geographic locations and their probability densities were estimated via the Wildlife Computers® data portal using the tag manufacturer’s proprietary software (WC-GPE3 [33], analytical approach and resultant fish tracks are reported in Perle et al. [13]). All analyses were conducted in the MATLAB computing environment [34].

### Identification of behavioral modes

Identification of dolphinfish behaviors potentially indicative of association (or lack of association) with floating objects relied upon *in situ* observations of dolphinfish behavior associated (and unassociated) with FADs in the Central Pacific, Southwestern Indian, and Western Indian Oceans as reported by Whitney and colleagues [26]. Specifically, dolphinfish spent on average 94.8% of their time in the upper 10 m of the water column when associated with fish aggregating devices (FADs), but only 29.4% of their time in the upper 10 m when not associated with floating objects.

Because these behavioral patterns were observed consistently across study regions and monitoring methods, we adopted the vertical distributions as behavioral indicators of periods when dolphinfish were associated and unassociated with any floating object. Lacking direct observation or estimation of the presence/absence of FADs or floating objects, we modified Whitney et al.’s [26] nomenclature of FAD-A (i.e., fish-aggregating-device associated) and FAD-U (i.e., fish-aggregating-device unassociated) to *p*FO-A (i.e., potential floating-object-associated) and *p*FO-U (potential floating-object-unassociated) respectively.

Dolphinfish daytime, nighttime, and 24-hour periods in the timeseries dataset were flagged as *p*FO-A if at least 90% of depth records were within the upper 10 m, or *p*FO-U if at least 50% of the depth records were deeper than 10 m. Day and night were classified by time of local sunrise and sunset, with the hour around sunrise and sunset excluded from the analysis. Periods in which dolphinfish spent between 50% and 90% of their time in the upper 10 m were times when the fish could not be confidently labeled as either associated or not associated with a floating object. These ‘gray areas’ were labeled as a third, “Surface-Oriented” behavior. Characterization of diurnal behavior was only conducted if the timeseries had sufficiently dense data (defined as a minimum of 50% of the recordings with gaps in the record not exceeding 3 hours over a 12-hour period). Otherwise, data-poor daytime, nighttime and 24-hour periods were labeled unknown.

### Analysis of behavioral modes

The prevalence of *p*FO-A behavior was evaluated with respect to fish size, region, and time of day (i.e., day or night). Size categories (small and large) were delineated based on the bimodal distribution of sizes of recaptured fish with an antimode at 100 cm (Table 1). Summary statistics are presented as the median [first quartile, third quartile]. We consider each fish to be an independent replicate due to the differences in the timing and locations of deployments as evidenced by the tracks. Statistical summaries for a group (e.g., OAX fish versus WBC fish) represent the distributions of the median value per fish in each group to avoid pseudo-replication and autocorrelation within the incomplete timeseries recovered from each fish. Likewise, to assess significant differences between groups, median values for each fish were compared using U = Test (Mann-Whitney, Gibbons [35]) such that no single fish tracking dataset would skew the outcomes.

The oceanography associated with *p*FO-A and *p*FO-U behaviors was investigated to assess whether each behavioral mode is linked with detectable oceanographic conditions. Thermal habitat was examined using both high-resolution (0.25° x 0.25°) blended analysis of daily sea surface temperature (SST) satellite imagery (OISSTv2 [36]) and the subsampled temperature timeseries data from individual tags. Tag derived SSTs were calculated as the average of temperature measurements taken in the upper 5 meters from each tag’s subsampled timeseries per twelve-hour period. Surface productivity for a given twelve-hour period was estimated based on the average of the daily, 4 km resolution, satellite-derived surface chlorophyll-a [37] concentrations intersecting the possible model-derived locations, weighted by each location’s probability distribution.

We also examined eddy kinetic energy, convergence, and vorticity derived from OSCAR one-third degree with a 5-day resolution current vectors [38] using the Gibbs SeaWater (GSW) Oceanographic Toolbox of TEOS-10 [39], to test if dolphinfish display more *p*FO-A behavior in convergent areas where floating objects are likely to accumulate [40,41] and in areas of high current velocities [25]. Satellite oceanographic data were matched with the fish’s daytime and nighttime locations, which have an estimated area of uncertainty of 38.8 km^2^ [23.3 km^2^, 68.2 km^2^] (calculated as the area that contains locations within the 95% confidence interval, WC-GPE3, [33]).

These oceanographic data were incorporated into a Principal Component Analysis (PCA) after log-transformation and min-max scaling. Results of the PCA were examined by behavioral mode, region, and fish size.

## Results

Constraints on tag deployments resulted in discrepancies in fish size and season between tagging regions. Fish in the OAX region were significantly larger (110 cm [107 cm, 113 cm]) than fish tagged in the WBC region (93 cm [90 cm, 95 cm], Mann-Whitney U-Test, p < 0.001, Table 1). Deployments in the OAX region took place during the winter months (Jan–Apr) while deployments in the WBC region occurred during summer and fall months (Jul–Nov, Table 1). The timing of deployments coincides with seasonal changes in regional currents that increase the prevalence of natural floating objects originating from kelp forests in the WBC and the forests and river deltas in the OAX (Fig 1) [30].

Independent of deployment region, all dolphinfish exhibited both surface-oriented behavior (SO) and potential floating-object-associated (*p*FO-A) behavior, and all but four dolphinfish (83%) exhibited potential floating-object-unassociated (*p*FO-U) behavior (Table 2). Despite the variance in fish length between regions, the overall size range of the dolphinfish in this study (85 cm– 118 cm, Table 1) is consistent with the size range of those tagged by Whitney et al. (58 cm– 125 cm FL) [26]. Diving behaviors were classified for 975 diurnal periods and 389 24-hr periods for 23 dolphinfish with deployments ranging from 7 to 68 days and a median deployment length of 27 days (Tables 1 and S1).

**Table 2:** Number of diurnal and 24-hr periods per behavioral mode for each fish. Percentages are given in parentheses.

Region	Fork Length (cm)	Sex	*p*FO-A Behavior			*p*FO-U Behavior		
			Days	Nights	24-hr	Days	Nights	24-hr
OAX	103	Male	5 (13%)	0 (0%)	0 (0%)	28 (72%)	35 (95%)	21 (81%)
OAX	103	Male	11 (92%)	1 (9%)	2 (17%)	0 (0%)	3 (27%)	0 (0%)
OAX	107	Male	17 (46%)	5 (17%)	4 (14%)	10 (27%)	8 (27%)	6 (21%)
OAX	108	Female	16 (55%)	3 (12%)	4 (17%)	5 (17%)	12 (46%)	6 (26%)
OAX	110	Male	25 (68%)	0 (0%)	1 (3%)	4 (11%)	34 (81%)	7 (23%)
OAX	110	Male	2 (25%)	0 (0%)	0 (0%)	0 (0%)	7 (100%)	5 (63%)
OAX	113	Female	9 (22%)	4 (13%)	4 (15%)	14 (34%)	11 (35%)	7 (26%)
OAX	115	Female	6 (50%)	0 (0%)	1 (8%)	2 (17%)	9 (82%)	7 (58%)
OAX	118	Male	11 (35%)	4 (13%)	7 (28%)	10 (32%)	4 (12%)	5 (20%)
WBC	85	Female	25 (93%)	23 (72%)	6 (55%)	0 (0%)	0 (0%)	0 (0%)
WBC	88	Male	7 (58%)	6 (46%)	7 (44%)	0 (0%)	3 (23%)	0 (0%)
WBC	89	Female	8 (62%)	2 (17%)	9 (82%)	0 (0%)	2 (17%)	0 (0%)
WBC	90	Female	9 (39%)	7 (30%)	5 (42%)	4 (17%)	11 (48%)	0 (0%)
WBC	90	Male	6 (86%)	2 (29%)	2 (18%)	0 (0%)	0 (0%)	0 (0%)
WBC	91	Male	10 (83%)	7 (58%)	6 (27%)	1 (8%)	2 (17%)	9 (41%)
WBC	93	Female	3 (13%)	17 (63%)	3 (43%)	10 (42%)	4 (15%)	0 (0%)
WBC	93	Male	4 (57%)	3 (43%)	7 (58%)	1 (14%)	4 (57%)	1 (8%)
WBC	94	Female	16 (73%)	16 (64%)	6 (55%)	0 (0%)	1 (4%)	0 (0%)
WBC	95	Female	6 (75%)	6 (75%)	2 (29%)	0 (0%)	0 (0%)	0 (0%)
WBC	95	Female	17 (61%)	16 (50%)	7 (88%)	0 (0%)	1 (3%)	0 (0%)
WBC	98	Female	16 (73%)	18 (78%)	15 (52%)	0 (0%)	0 (0%)	0 (0%)
WBC	100	Male	27 (90%)	5 (18%)	18 (86%)	0 (0%)	3 (11%)	0 (0%)
WBC	106	Male	6 (67%)	5 (63%)	5 (71%)	0 (0%)	1 (13%)	0 (0%)

**Fig 2:**
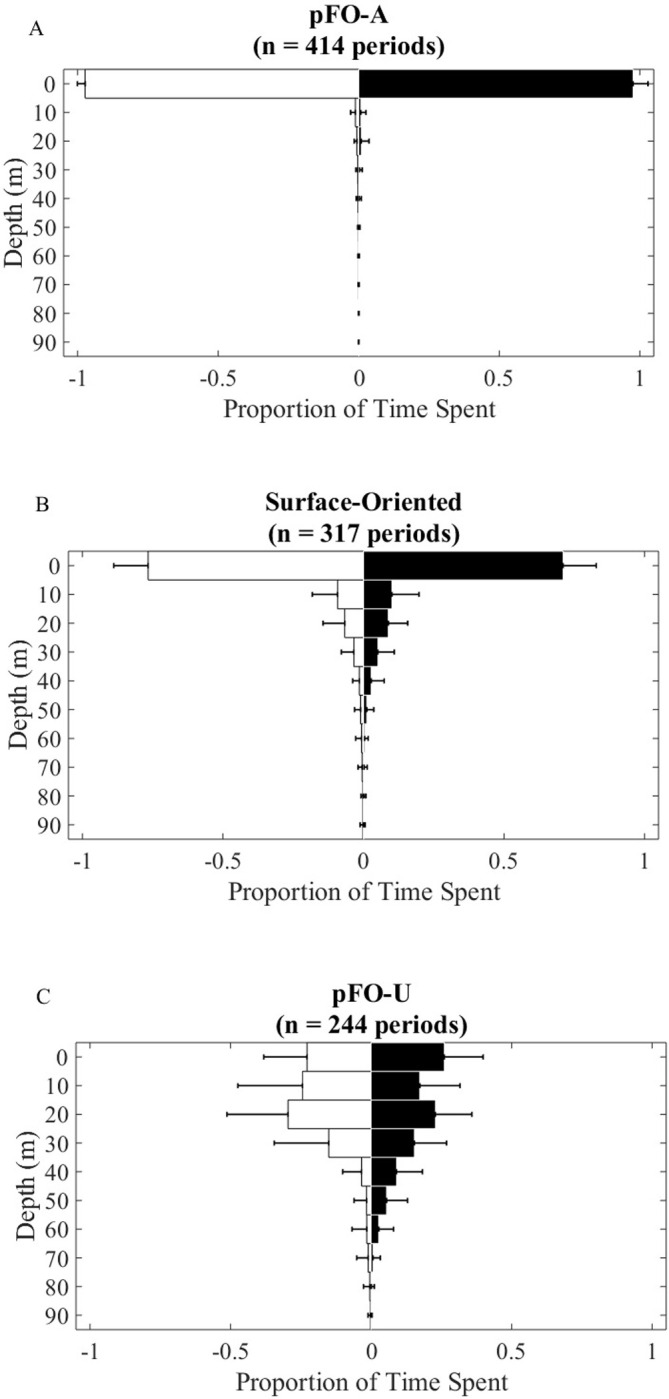
Day and night depth histograms for the 3 behavioral modes. (a) Potential floating-object-associated behavior, (b) Surface-Oriented behavior, and (c) Potential floating-object-unassociated diving behavior. The sample numbers are the total number of diurnal periods that each behavior was observed. White bars denote daytime while black bars denote nighttime distributions.

By definition, the general vertical distributions of *p*FO-A, surface-oriented, and *p*FO-U behaviors are characteristically distinct from one another (Fig 2). Diving behavior within these behavioral modes was found to be largely consistent across regions and by time of day. Median depth was not significantly different between regions or between day and night when fish were exhibiting *p*FO-A behavior (0.5 m [0.5 m, 1 m], region: *p =* 0.84, day vs night: *p* = 0.42) or *p*FO-U behavior (20.5 m [17.8 m, 28.5 m], region: *p =* 0.51, day vs night: *p* = 0.65). Within the *p*FO-U behavioral mode, maximum diving depth (49 m [38.9 m, 63.2 m]) and the variability in diving depth (measured as the IQR, 20.6 m [13.5 m, 28.8 m]) were also similar across regions (*p* = 1). However, during periods of *p*FO-A behavior, WBC fish dove to deeper maximum depths during the day (*p*FO-A: 33.3 m [26.3 m, 52.8 m]) than at night (*p*FO-A: 12.1 m [5.5 m, 27.5 m], Mann-Whitney U-Test, *p* = 0.002). The WBC’s daytime maximum diving depths during the *p*FO-A behavior were also deeper than those of the OAX fish (19 m [13.5 m, 30.5 m], *p* = 0.03). Also during daytime periods of *p*FO-A behavior, WBC fish were more variable in their diving than OAX fish (WBC: 33.3 m [26.3 m, 52.8 m]; OAX: 19 m [13.5 m, 30.5 m]), indicative of more active vertical movements in the WBC than in the OAX when exhibiting *p*FO-A behavior.

**Fig 3:**
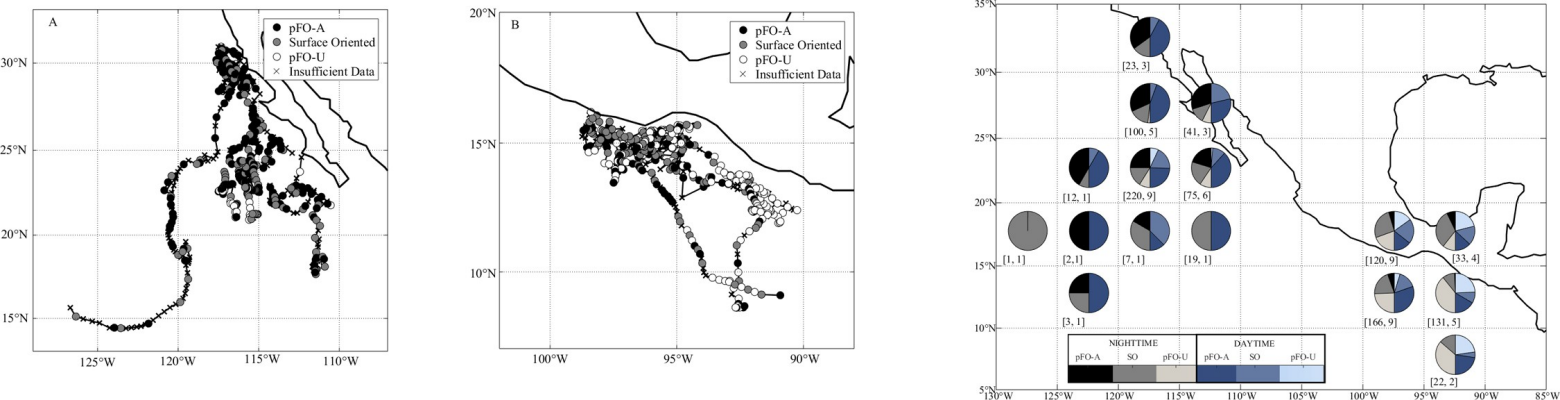
Distribution and proportion of potential floating-object-associated behavior. Behavioral changes along tracks in the (a) WBC and (b) OAX regions. (c) Spatial differences in the frequency of behavioral modes. Sample sizes are in brackets for each 5-degree grid (number of 12-hour periods, number of fish).

There was significant variability in the frequency of *p*FO-A behavior, with less *p*FO-A behavior occurring in the OAX region than in the WBC region (Fig 3 and Table 3). Regional differences in behavior are inevitably linked with regional differences in fish size–with the exception of two large fish in the WBC. There was no significant difference in the proportion of *p*FO-A or *p*FO-U behavior between males and females (Mann-Whitney U-Test) during the day (*p*FO-A: *p* = 0.73; *p*FO-U: *p* = 0.97), at night (*p*FO-A: *p* = 0.10; *p*FO-U: *p* = 0.23), or during a 24 hour period (*p*FO-A: *p* = 0.31; *p*FO-U: *p* = 0.97).

**Table 3:** Proportion of *p*FO-A and *p*FO-U behavior across the region and by size. Percentages shown are the median [first quartile, third quartile] of individual fish proportions of time spent in the behavior throughout the duration of their deployments.

	N (Fish)	Fork Length (cm)	*p*FO-A			*p*FO-U		
			Day (%)	Night (%)	24-Hr (%)	Day (%)	Night (%)	24-Hr (%)
**WBC**	**14**	**93 [90,95.8]**	**71 [58, 83]**	**48 [29, 64]**	**48 [29, 71]**	**0 [0, 8]**	**9 [0, 23]**	**0 [0, 0]**
FL < 100 cm	12	92 [89.5, 94.8]	66 [58, 79]	54 [34, 68]	47 [28, 70]	0 [0, 11]	9 [0, 20]	0 [0, 0]
FL ≥ 100 cm	2	103 [100,106.8]	82 [73, 91]	21 [17, 25]	58 [44, 71]	0 [0,0]	15 [4, 25]	0 [0, 0]
**OAX**	**9**	**110 [106,113.8]**	**47 [25, 57]**	**9 [0, 13]**	**14 [3, 17]**	**18 [8, 32]**	**46 [28, 86]**	**26 [21, 59]**
FL < 100 cm	0	-	-	-	-	-	-	-
FL ≥ 100 cm	9	110 [106,113.8]	47 [25, 57]	9 [0, 13]	14 [3, 17]	18 [8, 32]	46 [28, 86]	26 [21, 59]
**All**	**23**	**98 [91.5,107.8]**	**61 [41, 74]**	**25 [12, 56]**	**28 [15, 54]**	**0 [0, 18]**	**23 [4, 47]**	**0 [0, 25]**
FL < 100 cm	12	92 [89.5, 94.5]	66 [58, 79]	54 [37, 68]	47 [28, 70]	0 [0, 11]	9 [0, 20]	0 [0, 4]
FL ≥ 100 cm	11	108 [103.8, 112.3]	50 [28, 71]	12 [0, 16]	15 [5, 25]	17 [0, 30]	35 [26, 82]	23 [5, 50]

**Fig 4:**
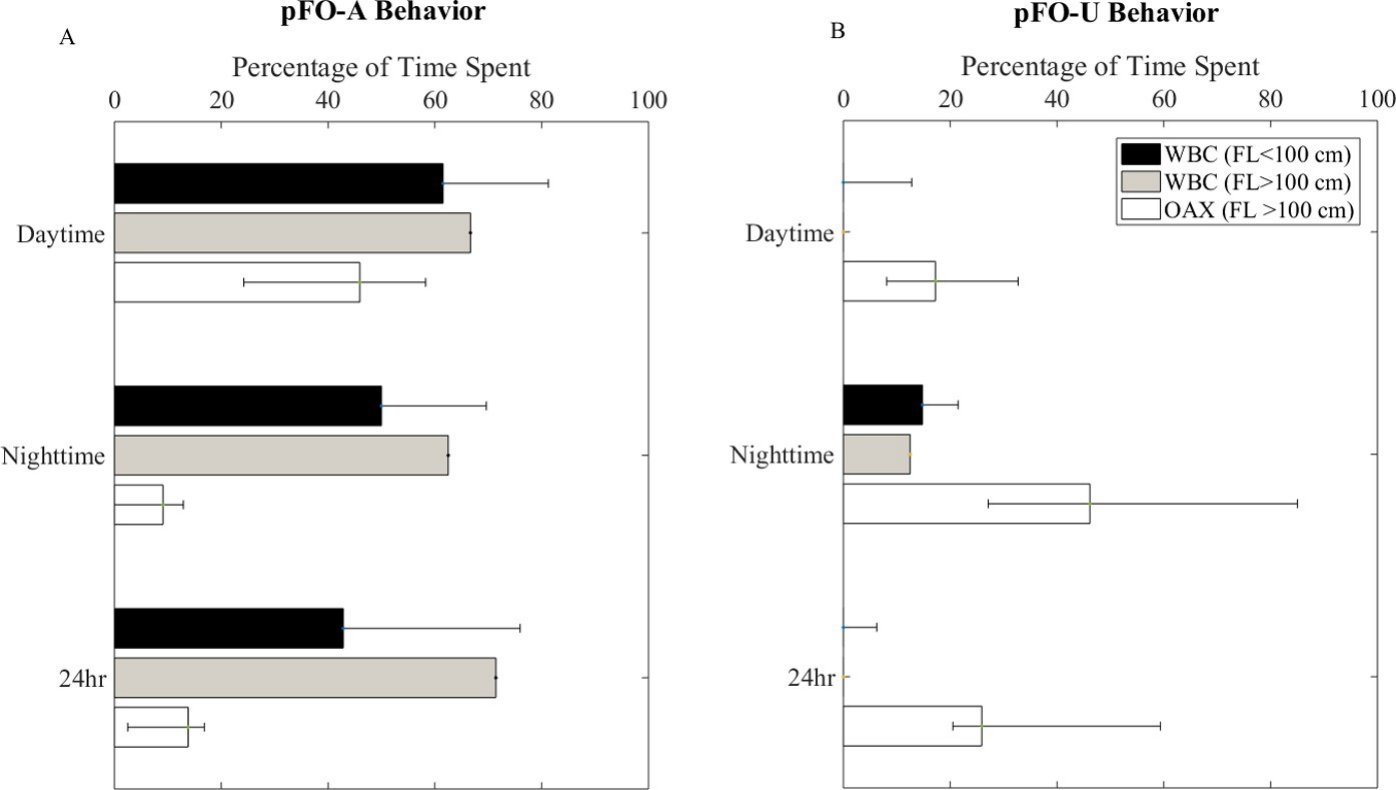
Size and Region comparison of frequency of (a) *p*FO-A and (b) *p*FO-U behaviors.

Fish in the OAX–all larger than 100 cm fork length–spent significantly less time in *p*FO-A than fish in the WBC–including the fish in the WBC larger than 100 cm fork length–during the day (Mann-Whitney U-Test, *p* = 0.04, at night (Mann-Whitney U-Test, *p* = 0.001) and over 24-hour periods (Mann-Whitney U-Test, p < 0.002, Table 2 and Fig 4A**).** Furthermore, fish in the OAX region spent more of their time exhibiting *p*FO-U behavior during the day (Mann-Whitney U-Test, p = 0.019), night (Mann-Whitney U-Test, p = 0.002), and over 24-hour periods (Mann-Whitney U-Test, p = 0.009, Table 3 and Fig 4B) than fish in the WBC region. Interestingly, the larger fish in the WBC significantly differed in their frequency of both *p*FO-A and *p*FO-U behaviors during the night periods from the OAX fish (Mann-Whitney U-Test, p = 0.04), but were not significantly different in their behavior relative to the other fish in the WBC (Mann-Whitney U-Test, p > 0.44 for both *p*FO-A and *p*FO-U behaviors during daytime, nighttime, and over 24 hour periods, Table 3 and Fig 4). Lastly, diurnal patterns in the frequency of *p*FO-A and *p*FO-U behavior were only observed in the OAX region, where fish tended to exhibit *p*FO-A behavior during the day and *p*FO-U behavior at night (Mann-Whitney U-Test, *p*FO-A: p < 0.001, *p*FO-U: p = 0.02, Table 3).

**Fig 5:**
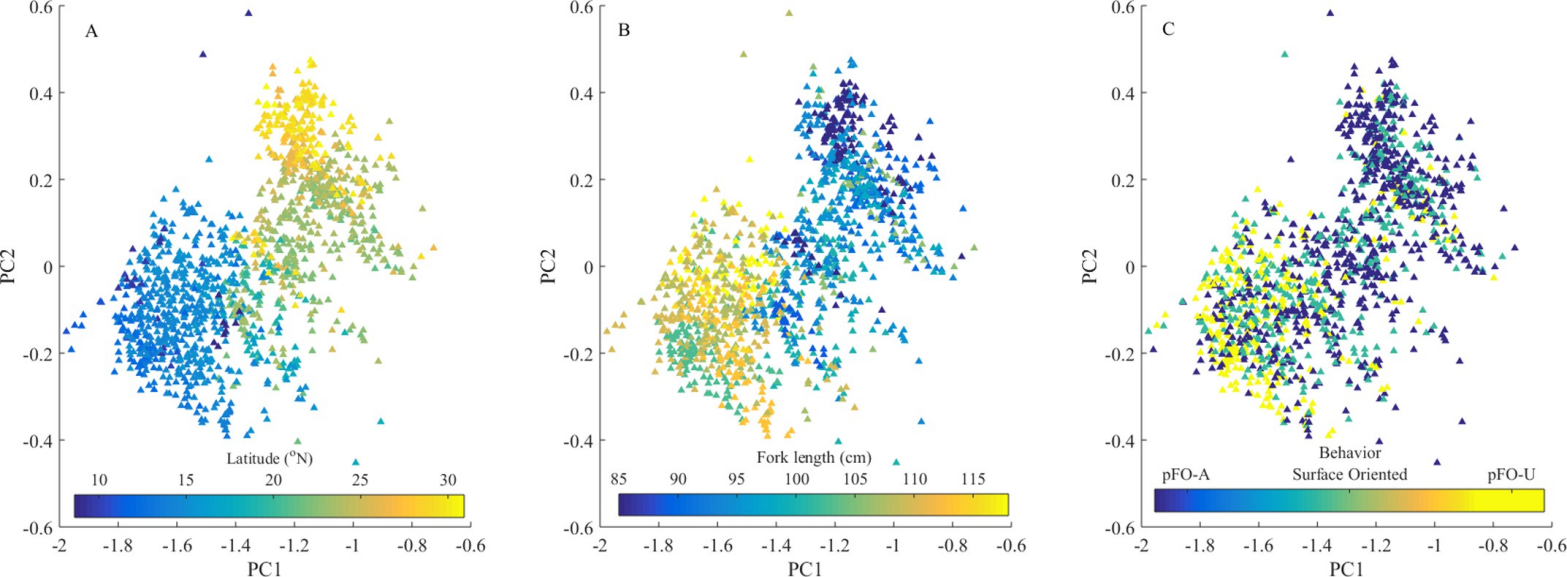
Principal component analysis of oceanographic conditions. Colors represent (a) latitude, (b) fish size, and (c) behavior.

As sampled the oceanographic environment differed significantly between WBC and OAX. These regional differences coincided with the different dolphinfish behaviors in our dataset. The OAX habitat was warmer, more productive, and had higher kinetic energy than the WBC habitat. (S2 Table). However, there were no systematic, within region oceanographic differences detected that underlaid either *p*FO-A and *p*FO-U behaviors (S2 Table). While Principal Component Analysis showed distinct groupings of the oceanographic and tagging datasets by region and size, the behavioral modes could be found across oceanographic environments (Fig 5 and S3 and S4 Tables).

## Discussion

This study uncovered regional differences in frequency and timing of dolphinfish behavioral modes indicative of dolphinfish association with floating objects (*p*FO-A). Fish off the coast of Oaxaca, Mexico (OAX) spent less time *p*FO-A than fish off the Pacific coast of Baja California, Mexico (WBC). The WBC fish spent most of their time exhibiting *p*FO-A behavior during both the daytime and at night; whereas the OAX fish spent significantly less time exhibiting *p*FO-A at night than during the day. Fish from both regions maintained similar median depths within each behavioral mode. However, WBC fish showed wider vertical distributions than the OAX fish when exhibiting *p*FO-A during the daytime.

Diurnal behavior while *p*FO-A is consistent with the findings of short-term, direct-observation studies of dolphinfish at floating objects and fish aggregating devices. Our finding that the frequency of potential floating-object-association was greater during the day than at night is consistent with Gooding and Magnuson’s findings that dolphinfish tended to recruit to FADs during the day rather than at night, suggesting that they may use visual cues to find FADs [42]. Our finding that dolphinfish dove deeper during the day than at night while exhibiting *p*FO-A behavior agrees with the findings of Whitney et al. [26] and Massutí et al. [43]. These deep dives are likely foraging excursions [43] and may occur during the day due to better visibility of the prey and the floating object. However, dolphinfish have been reported to return to manmade FADs kilometers away, suggesting that dolphinfish can use other, non-visual, cues for locating floating objects [25].

**Fig 6:**
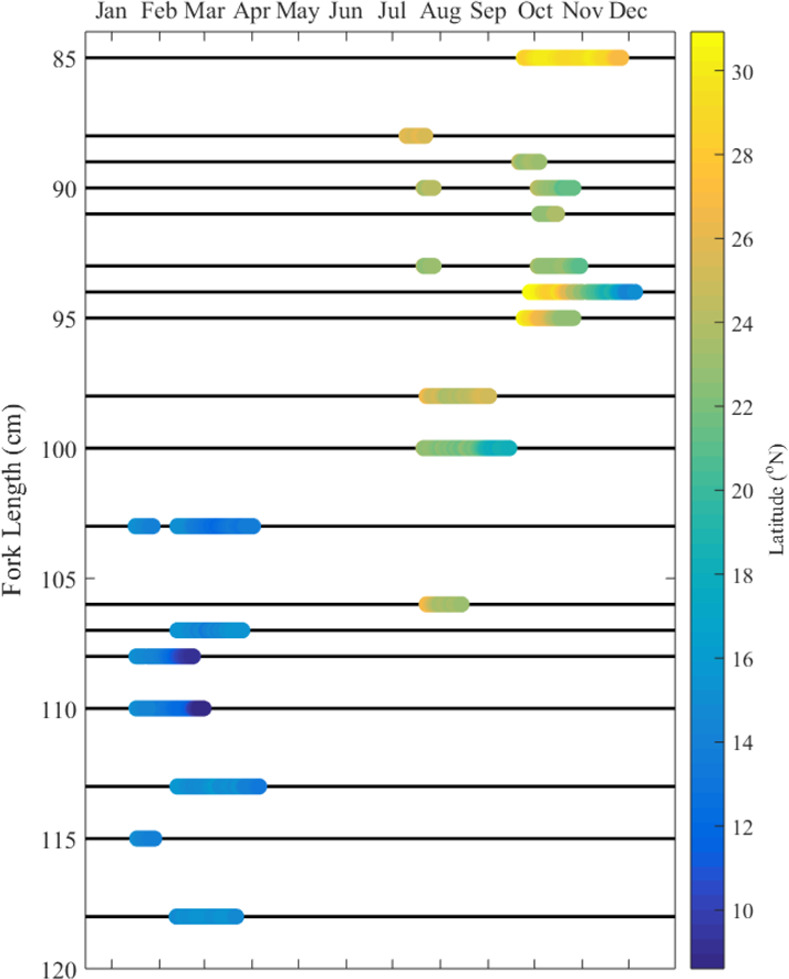
Season and fish size cofactors in dataset. Limitations in sampling (deployment lengths and fishing seasons) have led to larger fish in OAX during the winter to spring months and smaller fish in the WBC region during the summer to fall months.

While our study provides insight into the general extent and variability of the frequency and timing of floating-object-associated behavior, the tagging dataset cannot resolve the dynamics of these potential associations nor the potential causes of the observed regional differences in the frequency of this behavior. Associations with floating objects have been shown to vary in timescale from a few hours to several days, with some dolphinfish making excursions from floating objects for hours at a time [19,44]. As we were not able to detect short-term associations, this study cannot make claims about the duration of individual associations with a particular floating object and it is likely that our methods underestimate the instances of association with floating objects, particularly in cases where there were extensive excursions away from an object. Furthermore, the regional differences in the frequency of *p*FO-A behavior were confounded with regional differences in season and size/age, with significantly larger fish in the OAX region than in the WBC region (Fig 6). Because our regional difference in fish size has not been documented in the local purse seine fisheries [24], the selectivity of hook and line in WBC versus artisanal longline gear in OAX may have played a factor in the size discrepancy. The seasonal gaps in our dataset are due to both logistical difficulties in tagging outside of the fishing season in both regions [21] and the short duration of our tag deployments. Premature release of tags is a common issue in dolphinfish tagging studies (e.g., [27,45,46]) and could be attributed to either high rates of mortality or tag shedding or both.

Regional differences in fish size and age could account for the differences in behavior observed in this study. Dolphinfish caught and observed at floating objects are typically smaller than those caught or observed unassociated with floating objects. In the regional purse seine fishery, dolphinfish caught in log sets were significantly smaller than those caught in unassociated sets [24]. Furthermore, in the northwest Atlantic, larger dolphinfish did not associate with sargassum as often as smaller fish [47]. Floating objects may confer greater benefits to smaller dolphinfish as it is hypothesized that dolphinfish are more likely to use floating objects as shelter from predators and cleaning stations than as sources of food or shade [42]. On the other hand, the deep diving behavior (*p*FO-U) seen in this study is consistent with spawning behavior found in a recent study [45]; perhaps regional differences are attributable to larger, older fish spending more time spawning than smaller fish.

Although our sample size of large individuals in WBC is limited, it is noteworthy that the two fish larger than 100 cm in WBC were more similar in their frequencies of *p*FO-A and *p*FO-U behavior to the small fish in WBC than the large fish in the OAX region. Thus, regional differences in the number and type of floating objects in addition to the noted oceanographic differences may also contribute to the regional differences in *p*FO-A behavior. If the increased prevalence in *p*FO-A behavior is due to an increased abundance of floating objects, then dolphinfish in the WBC may have better foraging conditions with more refuge from predation and higher concentrations of prey. This difference in foraging conditions may explain why dolphinfish in the WBC have a larger length at maturation (L_50_ ~ 80 cm, [48]) than those in the OAX (L_50_ ~ 50 cm [14]) due to an increased growth rate while immature.

Sources of floating objects in the EPO in the Northern Hemisphere are kelp from the California Current, human debris (trash, discarded fishing gear), and terrestrial debris from river deltas [6]. In the WBC region, dolphinfish catch rates increase in areas with floating kelp mats found in convergent, warm waters [21,49]. Fishing on floating objects occurs in this region from June through November with peaks in August through September [21,30], corresponding with the senescence process of macroalgae at the end of summer [50] increasing the amount of floating macroalgae. Floating logs enter the current system via two local river outlets [30]. Log input likely peaks in June and July when the Costa Rica Coastal Current (CRCC) is the dominant surface current taking the debris NW along the coast [30]. Thus, the WBC receives logs from June through October [30]. Log-focused fishing is absent in the OAX region from May through January, but the reversal of the currents in the region and a dominant southward Countercurrent of Southern Mexico (CSM) in October through April brings concentrations of logs to the area. These log concentrations result in purse seine sets on logs beginning in January, with a peak in fishing effort and catch in February through April [24,30]. Thus, the tagging data in this study coincide with the seasonal peaks in the availability of floating objects and the presence of floating-object based fisheries in both regions (Fig 6).

In general, floating objects often appear in open ocean fronts or convergence zones [40,41], which occur at the submesoscale (order of 1–10 km) and are not detectable in the available ocean surface current satellite imagery [41]. This may account for our inability to link behavioral modes to surface oceanography. Although dolphinfish abundance at manmade FADs has been shown to increase with current speed [25], we saw a reduction in the *p*FO-A behavior with increasing current speeds across a regional scale. Thus, improvements in our ability to resolve submesoscale features through modeling efforts and finer scale in situ measurements will improve our ability to predict the presence/prevalence of floating objects.

Our study suggests dolphinfish (particularly small dolphinfish off the coast of Baja California, Mexico) spend a large amount of their time exhibiting *p*FO-A behavior, and this could influence their population structure and growth. Dolphinfish may travel with logs being transported from OAX to WBC via the Mexican Coastal Current, also known as the Western Mexican Current in the late spring—early fall months (May-October), or from WBC to OAX via the Counter Current of Southern Mexico during November and December [30,31]. While dolphinfish have been shown to move between these regions, the extent of this mixing remains unquantified and is not seen in our data [13]. While floating objects move around the EPO, they are targets of 11 nations whose fisheries catch dolphinfish on FAD or log sets [24]. Floating objects are thus important components to dolphinfish life history and international fishery management efforts. As dolphinfish become more prevalent in the global seafood trade [51], understanding the role that floating objects play in their population structure, growth, and catchability is essential to improving fishery management efforts.

## Supporting information

S1 Table Data availability for behavioral mode classification. Percent of data available for diurnal and 24 hr periods where behavioral mode was classified.
(DOCX)

S2 Table Regional oceanography. Oceanographic characteristics in Oaxaca and Western Baja California regions for 12-hr FAD-A and FAD-U Behavior. *Denotes significance at *p* <0.005.
(DOCX)

S3 Table Principal component coefficients. Coefficients for the oceanographic variables used in the principal component analysis.
(DOCX)

S4 Table Principle component analysis. Principal component results of oceanographic environment by region, size, and behavior.
(DOCX)
