# Supplementary material for: Vertical distributions of dolphinfish (Coryphaena hippurus) in the Eastern Pacific Ocean suggest variability in potential associations with floating objects
Source: PLoS One. 2022 Nov 1;17(11):e0276873. doi: 10.1371/journal.pone.0276873 (PMC9624430; doi:10.1371/journal.pone.0276873)
Supplement: S1 Table — Percent of data available for diurnal and 24 hr periods where behavioral mode was classified. (DOCX) [file pone.0276873.s001.docx]

**S1 Table. Data Availability for Behavioral Mode Classification.** Percent of data available for diurnal and 24 hr periods where behavioral mode was classified.

| Region | Sex | Size | Percent of Daytime Data Available | | Percent of Nighttime Data Available | | Percent of 24hr Data Available | |
| --- | --- | --- | --- | --- | --- | --- | --- | --- |
|  |  |  | Median [25th, 75th] | Min - Max | Median [25th, 75th] | Min - Max | Median [25th, 75th] | Min - Max |
| WBC | Female | 93 | 1 [0.82, 1] | 0.65 - 1 | 1 [0.87, 1] | 0.76 - 1 | 0.92 [0.92, 1] | 0.75 - 1 |
| WBC | Female | 90 | 1 [1, 1] | 0.65 - 1 | 1 [1, 1] | 0.81 - 1 | 1 [0.92, 1] | 0.83 - 1 |
| WBC | Male | 91 | 1 [1, 1] | 0.83 - 1 | 1 [1, 1] | 0.82 - 1 | 1 [1, 1] | 0.92 - 1 |
| WBC | Female | 95 | 1 [1, 1] | 0.82 - 1 | 1 [0.90, 1] | 0.62 - 1 | 1 [0.96, 1] | 0.92 - 1 |
| WBC | Male | 88 | 1 [0.94, 1] | 0.85 - 1 | 1 [1, 1] | 0.79 - 1 | 1 [0.92, 1] | 0.83 - 1 |
| WBC | Male | 100 | 0.82 [0.74, 0.92] | 0.57 - 1 | 0.80 [0.63, 0.83] | 0.58 - 1 | 0.75 [0.58, 0.92] | 0.5 - 1 |
| WBC | Male | 106 | 0.84 [0.77, 0.91] | 0.55 - 1 | 0.65 [0.56, 0.73] | 0.55 - 1 | 0.75 [0.52, 0.75] | 0.5 - 0.83 |
| WBC | Female | 98 | 0.84 [0.76, 0.92] | 0.5 - 1 | 0.98 [0.78, 1] | 0.58 - 1 | 0.75 [0.65, 0.92] | 0.5 - 0.92 |
| WBC | Male | 93 | 1 [0.94, 1] | 0.75 - 1 | 1 [1, 1] | 0.79 - 1 | 1 [0.92, 1] | 0.83 - 1 |
| WBC | Male | 90 | 1 [1, 1] | 0.86 - 1 | 1 [1, 1] | 0.79 - 1 | 1 [0.94, 1] | 0.92 - 1 |
| OAX | Male | 103 | 1 [0.81, 1] | 0.53 - 1 | 0.83 [0.70, 1] | 0.52 - 1 | 0.92 [0.83, 1] | 0.5 - 1 |
| OAX | Male | 118 | 1 [0.82, 1] | 0.54 - 1 | 0.82 [0.72,1] | 0.54 - 1 | 0.83 [0.73, 0.94] | 0.5 - 1 |
| OAX | Male | 107 | 0.89 [0.80, 1] | 0.53 - 1 | 1 [0.82, 1] | 0.53 - 1 | 0.83 [0.75, 1] | 0.5 - 1 |
| OAX | Female | 113 | 1 [0.82, 1] | 0.55 - 1 | 0.91 [0.82, 1] | 0.55 - 1 | 0.83 [0.75, 0.92] | 0.58 - 1 |
| WBC | Female | 94 | 0.77 [0.59, 0.81] | 0.52 - 1 | 0.68 [0.57, 0.83] | 0.51 - 1 | 0.58 [0.5, 0.75] | 0.5 - 0.75 |
| WBC | Female | 85 | 0.74 [0.60, 0.78] | 0.54 - 1 | 0.80 [0.74, 0.91] | 0.52 - 1 | 0.67 [0.5, 0.81] | 0.5 - 0.92 |
| WBC | Female | 89 | 0.81 [0.79, 1] | 0.60 - 1 | 0.82 [0.79, 0.97] | 0.58 - 1 | 0.92 [0.71, 0.98] | 0.58 - 1 |
| WBC | Female | 95 | 0.94 [0.81, 1] | 0.56 - 1 | 1 [0.86, 1] | 0.76 - 1 | 0.92 [0.83, 1] | 0.58 - 1 |
| OAX | Male | 110 | 1 [0.83, 1] | 0.65 - 1 | 1 [0.88, 1] | 0.53 - 1 | 0.92 [0.92, 1] | 0.58 - 1 |
| OAX | Male | 110 | 1 [1, 1] | 1 - 1 | 1 [1, 1] | 1 - 1 | 1 [1, 1] | 1 - 1 |
| OAX | Male | 103 | 1 [1, 1] | 1 - 1 | 1 [1, 1] | 1 - 1 | 1 [1, 1] | 1 - 1 |
| OAX | Female | 115 | 1 [0.92, 1] | 0.82 - 1 | 1 [1, 1] | 0.83 - 1 | 1 [0.96, 1] | 0.83 - 1 |
| OAX | Female | 108 | 1 [0.73, 1] | 0.54 - 1 | 0.82 [0.82, 1] | 0.54 - 1 | 0.92 [0.83, 0.98] | 0.67 - 1 |
