# Supplementary material for: Vertical distributions of dolphinfish (Coryphaena hippurus) in the Eastern Pacific Ocean suggest variability in potential associations with floating objects
Source: PLoS One. 2022 Nov 1;17(11):e0276873. doi: 10.1371/journal.pone.0276873 (PMC9624430; doi:10.1371/journal.pone.0276873)
Supplement: S2 Table — Oceanographic characteristics in Oaxaca and Western Baja California regions for 12-hr FAD-A and FAD-U Behavior. *Denotes significance at p <0.005. (DOCX) [file pone.0276873.s002.docx]

**S2 Table. Regional Oceanography.** Oceanographic characteristics in Oaxaca and Western Baja California regions for diurnal pFO-A and pFO-U Behavior. *Denotes significance at *p* < 0.05 and ** denotes within region significance between behaviors at *p* <0.005.

|  | | Oaxaca | | | Western Baja California | | |
| --- | --- | --- | --- | --- | --- | --- | --- |
|  |  | All OAX | pFO-A | pFO-U | All WBC | pFO-A | pFO-U |
| Thermal Habitat | |  |  |  |  |  |  |
|  | SST | 28.2 [27.4, 29] | 28.4 [27.5, 29] | 28.4 [27.5, 29] | 22.3 [21.6, 23.6] | 22.2 [21.5, 23.4] ** | 23.0 [22.5, 23.4]** |
| Productivity | |  |  |  |  |  |  |
|  | CHL | 0.21 [0.08, 0.43] | 0.23 [0.08, 0.41] | 0.22 [0.14, 0.42] | 0.06 [0.00, 0.11] | 0.06 [0.00, 0.11] | 0.07 [0.00, 0.08] |
| Physical | |  |  |  |  |  |  |
|  | EKE | 0.03 [0.02, 0.07] | 0.03 [0.02, 0.07]** | 0.05 [0.02, 0.11]** | 0.01 [0.00, 0.01] | 0.01 [0.00, 0.01]** | 0.00 [0.00, 0.01]** |
|  | Max Conv (x10-6) | 5.48 [2.46, 8.96] | 4.98 [2.16, 8.41]** | 6.78 [2.49, 10.9]** | 4.17 [1.51, 7.01] | 3.86 [0.99, 6.99] | 2.30 [1.22, 3.75] |
|  | Max Vort (x10-7) | 3.06 [1.13, 5.83] | 2.79 [1.09, 5.83]** | 4.62 [1.24, 6.64]** | 0.96 [0.25, 2.61] | 0.72 [0.09, 2.46]** | 0.60 [0.17, 0.94] ** |
