# Supplementary material for: Vertical distributions of dolphinfish (Coryphaena hippurus) in the Eastern Pacific Ocean suggest variability in potential associations with floating objects
Source: PLoS One. 2022 Nov 1;17(11):e0276873. doi: 10.1371/journal.pone.0276873 (PMC9624430; doi:10.1371/journal.pone.0276873)
Supplement: S3 Table — Coefficients for the oceanographic variables used in the principal component analysis. (DOCX) [file pone.0276873.s003.docx]

**S3 Table. Principal Component Coefficients.** Coefficients for the oceanographic variables used in the principal component analysis.

|  | PC1 | PC2 |
| --- | --- | --- |
| SST | -0.4146 | -0.8671 |
| Chl a | -0.3646 | 0.1503 |
| Max Convergence | -0.5157 | 0.2890 |
| Max Vorticity | -0.4725 | 0.3740 |
| EKE | -0.4539 | -0.0464 |
