# Supplementary material for: Vertical distributions of dolphinfish (Coryphaena hippurus) in the Eastern Pacific Ocean suggest variability in potential associations with floating objects
Source: PLoS One. 2022 Nov 1;17(11):e0276873. doi: 10.1371/journal.pone.0276873 (PMC9624430; doi:10.1371/journal.pone.0276873)
Supplement: S4 Table — Principal component results of oceanographic environment by region, size, and behavior. (DOCX) [file pone.0276873.s004.docx]

|  | PC1 | PC2 |
| --- | --- | --- |
| Region |  |  |
| OAX | -1.57 [-1.67, -1.46] | -0.12 [-0.20, -0.03] |
| WBC | -1.17 [-1.24, -1.08] | 0.17 [0.04, 0.26] |
| Size |  |  |
| Larger than 100 cm | -1.55 [-1.66, -1.42] | -0.10 [-0.20, -0.01] |
| Smaller than 100 cm | -1.17 [-1.25, -1.08] | 0.17 [0.06, 0.26] |
| Behavior |  |  |
| FAD-U | -1.59 [-1.69, -1.41] | -0.10 [-0.20, 0.02] |
| FAD-A | -1.22 [-1.45, -1.11] | 0.10 [-0.10, 0.23] |

**S4 Table. Principle Component Analysis.** Principal component results of oceanographic environment by region, size, and behavior.
